# Incubation period, clinical and lung CT features for early prediction of COVID-19 deterioration: development and internal verification of a risk model

**DOI:** 10.1186/s12890-022-01986-0

**Published:** 2022-05-12

**Authors:** Hongbing Peng, Chao Hu, Wusheng Deng, Lingmei Huang, Yushan Zhang, Baowei Luo, Xingxing Wang, Xiaodan Long, Xiaoying Huang

**Affiliations:** 1 Department of Pulmonary and Critical Care Medicine, Loudi Central Hospital, No. 51, Changqing Middle Street, Loudi, 417000 People’s Republic of China; 2 Department of Pulmonary and Critical Care Medicine, Xiangtan Central Hospital, No. 120, Road Heping, Distract Yuhu, Xiangtan, 411100 People’s Republic of China; 3 Department of Pulmonary and Critical Care Medicine, Shaoyang Central Hospital, No. 36 Hongqi Road, Shaoyang, 422000 People’s Republic of China; 4 Department of Pulmonary and Critical Care Medicine, The First People’s Hospital of YueYang, No. 39 Dongmaoling Road, Yueyang, 414000 People’s Republic of China; 5 Department of Pulmonary and Critical Care Medicine, Huaihua First People’s Hospital, No. 144, Jinxi South Road, Huaihua, 418000 People’s Republic of China; 6 Department of Respiratory Medicine, Shuangfeng County People’s Hospital, 238 Shuyuan Road, Shuangfeng County, 417007 People’s Republic of China; 7 Department of Infection, Loudi Central Hospital, No. 51, Changqing Middle Street, Loudi, 417000 People’s Republic of China; 8 Department of Urology Surgery, Loudi Central Hospital, No. 51, Changqing Middle Street, Loudi, 417000 People’s Republic of China; 9 Department of Pulmonary and Critical Care Medicine, Loudi Central Hospital, No. 51, Changqing Middle Street, Loudi, 417000 People’s Republic of China

**Keywords:** Prediction model, COVID-19, Incubation period, Semi-quantitative CT score, Change-in-estimate (CIE), Deterioration

## Abstract

**Background:**

Most severe, critical, or mortal COVID-19 cases often had a relatively stable period before their status worsened. We developed a deterioration risk model of COVID-19 (DRM-COVID-19) to predict exacerbation risk and optimize disease management on admission.

**Method:**

We conducted a multicenter retrospective cohort study with 239 confirmed symptomatic COVID-19 patients. A combination of the least absolute shrinkage and selection operator (LASSO), change-in-estimate (CIE) screened out independent risk factors for the multivariate logistic regression model (DRM-COVID-19) from 44 variables, including epidemiological, demographic, clinical, and lung CT features. The compound study endpoint was progression to severe, critical, or mortal status. Additionally, the model's performance was evaluated for discrimination, accuracy, calibration, and clinical utility, through internal validation using bootstrap resampling (1000 times). We used a nomogram and a network platform for model visualization.

**Results:**

In the cohort study, 62 cases reached the compound endpoint, including 42 severe, 18 critical, and two mortal cases. DRM-COVID-19 included six factors: dyspnea [odds ratio (OR) 4.89;confidence interval (95% CI) 1.53–15.80], incubation period (OR 0.83; 95% CI 0.68–0.99), number of comorbidities (OR 1.76; 95% CI 1.03–3.05), D-dimer (OR 7.05; 95% CI, 1.35–45.7), C-reactive protein (OR 1.06; 95% CI 1.02–1.1), and semi-quantitative CT score (OR 1.50; 95% CI 1.27–1.82). The model showed good fitting (Hosmer–Lemeshow goodness, X^2^(8) = 7.0194, *P* = 0.53), high discrimination (the area under the receiver operating characteristic curve, AUROC, 0.971; 95% CI, 0.949–0.992), precision (Brier score = 0.051) as well as excellent calibration and clinical benefits. The precision-recall (PR) curve showed excellent classification performance of the model (AUC_PR_ = 0.934). We prepared a nomogram and a freely available online prediction platform (https://deterioration-risk-model-of-covid-19.shinyapps.io/DRMapp/).

**Conclusion:**

We developed a predictive model, which includes the including incubation period along with clinical and lung CT features. The model presented satisfactory prediction and discrimination performance for COVID-19 patients who might progress from mild or moderate to severe or critical on admission, improving the clinical prognosis and optimizing the medical resources.

**Supplementary Information:**

The online version contains supplementary material available at 10.1186/s12890-022-01986-0.

## Introduction

The global pandemic of COVID-19 caused by the severe acute respiratory syndrome coronavirus (SARS-COV-2) has started in December 2019 and it has been around for 2 year now [1, 2]. As of May 9, 2021, the World Health Organization (WHO) reported that more than 1.5 billion infected people worldwide, and more than 3.29 million deaths occurred. The fatality rate in the early stage of the disease is more than 7.0% [3, 4]. This pandemic poses a significant threat to global health.

The reported clinical outcomes of different severity grades are heterogeneous, and the mild and moderate cases often rely on their immune ability to recover [5, 6]. However, most severe or critical COVID-19 patients are asymptomatic at the initial stage of onset, and the median time from onset to sepsis is 10.0 days [interquartile range (IQR) 7.0–14.0] [7]. Early screening and active intervention in critical patients could reduce mortality [8]. A deterioration model of early prediction of COVID-19 progression from mild or moderate to severe, critical or mortal might help front-line clinicians to optimize the patient triage and develop appropriate treatment strategies.

Many multivariate clinical prognostic models for predicting the deterioration of COVID-19 have been published [9–13]. The predictors mainly include demographic, clinical, and laboratory factors. However, the included factors are rarely involved in epidemiology and chest imaging features, such as the incubation period. Although the incubation period was the key feature and essential basis in the study of epidemic control and prediction [14], there were relatively few studies on the deterioration of COVID-19. Early studies found that the incubation period of travelers to Hubei was shorter than that of non-travelers [15]. The incubation period was negatively correlated with the severity of COVID-19 [14]. Furthermore, high CT scores characterized severe/critical COVID-19 pneumonia [16, 17]. Further research is still needed to determine whether epidemiology and lung CT features can improve the predictive ability of the deterioration model.

In this study, we present a prediction model of COVID-19 (DRM-COVID-19) with epidemiological, clinical, and pulmonary CT characteristics, which could predict the risk of COVID-19 deterioration on admission. The COVID-19 epidemic is still raging globally, and we hope our model can provide convenience for front-line clinicians to make individualized treatment decisions, reduce the deterioration and optimize the use of medical resources.

## Methods

### Participants, compound endpoint, and design

In this work, we established a retrospective cohort study on patients from five hospitals in Hunan Province (Loudi Central Hospital, Xiangtan Central Hospital, Yueyang First People's Hospital, Shaoyang Central Hospital, and Huaihua First People's Hospital) between January 11 and February 28, 2020. Considering the particularity of the COVID-19 disease, all the data were collected anonymously and retrospectively, protecting the patients' privacy. The ethics committee of the Loudi central hospital approved this study and waived the need for written informed consent for the new infectious diseases. Our study followed the World Medical Association’s Declaration of Helsinki.

According to the World Health Organization (WHO) living guidance of COVID-19 [18], 246 patients diagnosed with COVID-19 by pharyngeal or nasopharyngeal swabs were enrolled in this study. According to the WHO guidelines [18], the cases were classified as mild, moderate, severe, or critical [including acute respiratory distress syndrome (ARDS), sepsis, septic shock]. As the primary outcome, deterioration refers to the progression from mild or moderate to severe, critical or mortal [9, 10].

**Fig. 1:**
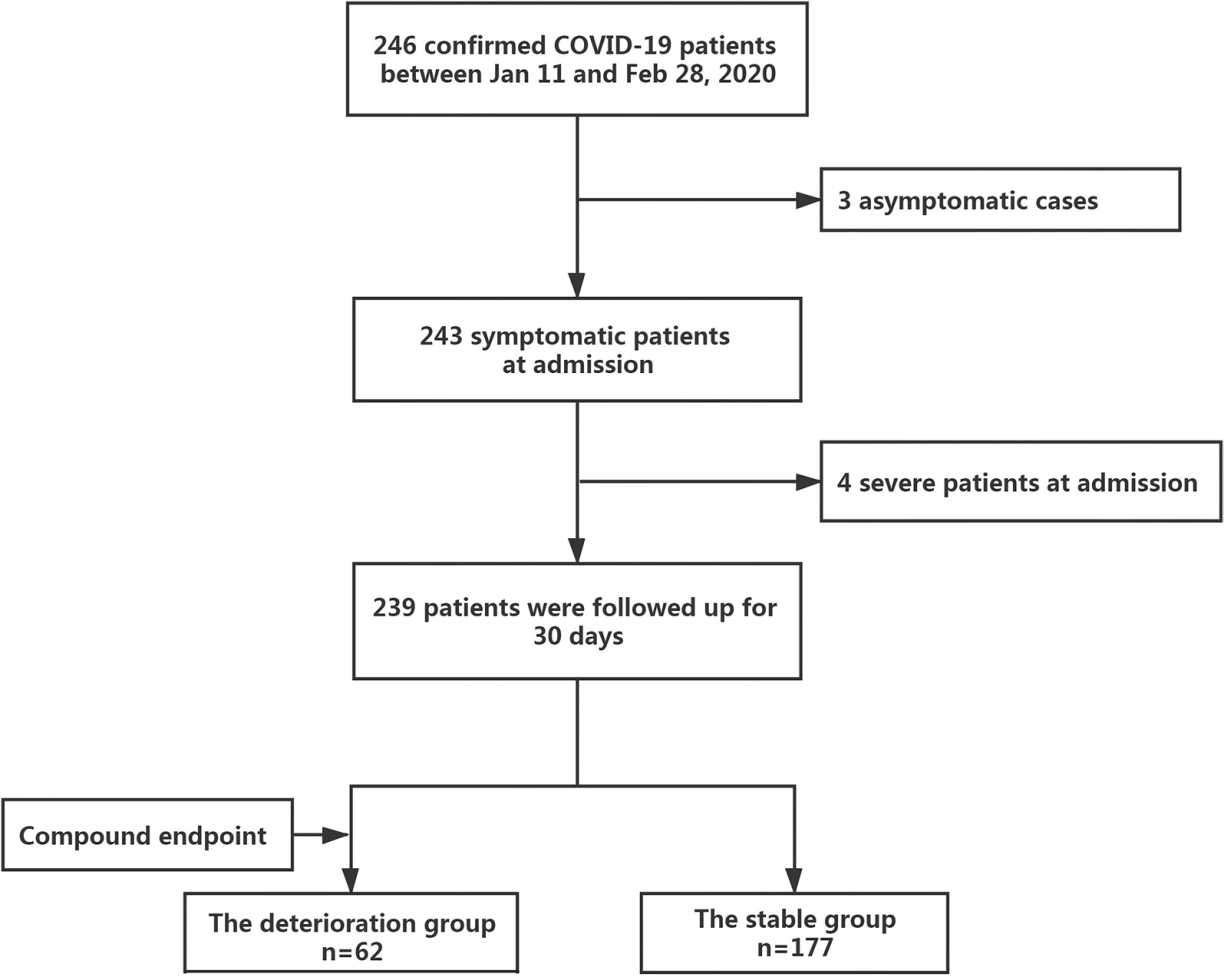
The flow chart of this study

We excluded three COVID-19 patients who were asymptomatic since onset and four patients who were diagnosed as severe or critical on admission. Then, the remaining 239 patients were followed up for 30 days, among which 62 patients worsened and reached the primary outcome, and were included in the deterioration group. Hence, 177 patients represented the stable group (Fig. 1).

### Data collection

The collected data included the demographics, comorbidities, clinical symptoms, laboratory results, and imaging data, all of which were cross-verified by two experienced physicians from the electronic health records (ERH) in each COVID-19 treatment center. Clinical symptoms, laboratory results, and image data were collected at the day of admission.

The demographic and epidemiological data included the following: age, gender, Wuhan origin (living in Wuhan, traveling or taking public transport through Wuhan), and the incubation period (the time from the first exposure to onset [14]), and the length of hospital day. Comorbidities data included the following: number of comorbidities, coronary heart disease, hypertension, endocrine system disease (diabetes, obesity, hyperlipidemia, or hyperthyroidism), chronic lung disease (chronic obstructive pulmonary disease, asthma, chronic bronchitis, emphysema, or bronchiectasis), malignant tumor, and chronic digestive system diseases (viral hepatitis, liver cirrhosis, fatty liver, or drug-induced liver injury). The symptoms at admission included: fever (the highest body temperature before admission), cough, dyspnea, headache, dizziness, muscle pain, fatigue, and gastrointestinal symptoms. The laboratory data included: white blood cell count, neutrophil count, lymphocyte count, neutrophil/lymphocyte ratio, platelet count, hemoglobin, D-dimer, albumin, total bilirubin, direct bilirubin, creatine kinase, creatine kinase isoenzyme, lactate dehydrogenase, myoglobin, urea nitrogen, creatinine, blood glucose, C-reactive protein, and procalcitonin. As for the imaging data, a semi-quantitative scoring system was used to evaluate the score of each affected lobe as follows: 0, no involvement; 1, less than 5%; 2, 5–25%; 3, 26–49%; 4, 50–75%; 5, more than 75%; then, the scores were summed to obtain the total pulmonary involvement score [16, 17]. Two radiologists independently scored the image analysis according to the semi-quantitative score system, and then the average value was calculated and used.

### Statistical methods and variable selection

The baseline data table presented continuous variables as median (IQR) and categorical variables as n (%). The Mann–Whitney U test, Chi-square test, or Fisher's exact test were used to compare the differences between the stable group and deterioration group when appropriate. Statistical analysis was performed using the R software (version 3.6.3, R Foundation). All the cases were enrolled in the variable selection and risk model development. All the required diseases information and variable values must be collected at admission. In case of outpatients, there must be no missing values. The L1-penalized LASSO regression was applied to reduce the data dimensionality to avoid potential collinearity and overfitting among variables. The best lambda value was selected in LASSO regression using tenfold cross-validation. Under the lambda compression (lambda.1se), the variables with small regression coefficients were directly compressed to 0 to eliminate the corresponding variables. Finally, only the most robust predictors were retained in the regression model. The LASSO regression was completed with the glmnet package [19].

### Risk model development and internal validation

We started by analyzing the variables selected by LASSO regression through single-factor regression. According to the previously reported characteristic variables [4, 20], we followed the change-in-estimate (CIE) [21, 22] approach to simplify the complete model. CIE is a data-driven independent variable screening method. CIE removes variables from the multivariable regression model that contribute less than 0.1 (10%) to change in odds ratio (OR) of essential variables [22]. A binary logistic regression model was established using the R package "caret" [23] and evaluated by the Hosmer–Lemeshow test. We performed internal validation with 1000 times of bootstrap resampling by counting R-squared (R2) and c-statistic for the evaluation and used nomogram and network calculator to visualize the predictive ability of DRM-COVID-19 using the DynNom [24] package.

### Evaluating the risk model

The binary logistic regression prediction models were separately established for the incubation period, clinical (clinical symptoms and laboratory data) or CT score. The distinguishing ability between the above-mentioned prediction models and DRM-COVID-19 was compared by using the receiver operating characteristic (ROC) curve. The model sensitivity (true positive rate) and specificity (true negative rate) were evaluated in the ROC curve, and the area under the receiver operating characteristic curve (AUC) was calculated. Due to the imbalance in our data, the risk model was further evaluated by the metrics of accuracy, precision, recall, F1 score, and precision-recall curve (PRC). We wanted our risk model to avoid a missed diagnosis of deteriorating risk patients. Meanwhile, we wanted to improve the prediction precision while ensuring a good recall. Since the values of precision and recall could not be simultaneously high, the comprehensive evaluation index of F1 score was introduced. We used the R package "modEvA" to generate the curve of the four evaluation indicators and calculate the number of cases to construct the confusion matrix [25]. We assessed the model accuracy by the logistic calibration curve, which visually demonstrated the consistency between the predicted and real results of DRM-COVID-19 (rms packets) [26]. Meanwhile, the calibration curve included c-statistic (ROC), R2, and Brier score to evaluate the model performance. In order to assess the clinical usefulness of DRM-COVID-19, we used different decision thresholds (Pt 10–95%) to establish a decision curve evaluating the net benefit of DRM-COVID-19 based on the following equation [27]:

$${\text{Net}}\;{\text{benefit}} = \frac{{{\text{True}}\;{\text{Positive}}\;{\text{Count}}}}{{\text{n}}} - \frac{{{\text{False}}\;{\text{Positive}}\;{\text{Count}}}}{{\text{n}}}\left( {\frac{{{\text{Pt}}}}{{1 - {\text{Pt}}}}} \right).$$

The clinical impact curve shows the practical clinical value.

### Secondary outcome analyses

The analysis of secondary outcomes included two aspects. First of all, the developed DRM-COVID-19 was used to calculate the predicted model of each case in the dataset, including stable and deterioration (severe, critical, and mortality) groups. The R package "ggstatsplot" [28] was used to visualize the data distribution, and the Kruskal–Wallis test was performed. Finally, the stable group was compared with other groups. Second, we applied univariate COX proportional hazard regression (Cox regression) to estimate successively the correlation between the predicted value of DRM-COVID-19 and exacerbation within 15 days, and the length of hospital stay. According to the optimal cutoff value of the predicted value, the patients were divided into the low-risk group (≤ 0.263) and high-risk group (> 0.263). The Kaplan–Meier method estimated the time-event curve and compared it with the bilateral log-rank test.

## Results

### Patients' demographics and characteristics

The data of 239 COVID-19 patients were used to train the deterioration risk model. Among these, 62 patients (25.9%) reached the study compound endpoint, including 42/62 severe, 18/62 critical (admission to the intensive care unit (ICU), mechanical ventilation), and 2/62 eventually died; the remaining patients were discharged. The deterioration group had the following characteristics compared with the stable group: older age (median 54.8 vs. 41.5), slightly more men (32/62 vs. 30/62), fever (> 38.0, 66.1% vs. 37.3%), dyspnea (74.2% vs. 9.0%), cough (90.3% vs. 72.3%) and fatigue (54.1% vs. 27.1%), respectively.

Similarly, the patients in the deterioration group were more likely to have comorbidities compared with the stable group (61.3% vs. 22.6%), especially endocrine and metabolic diseases (29.0% vs. 6.78%), hypertension (30.6% vs. 8.47%), respiratory system diseases (12.9% vs. 4.52%) and coronary heart disease (11.3% vs. 2.82%) respectively. There was only one case of the malignant tumor. The study noted that the patients with a short incubation period also had a higher risk of deterioration than those in the stable group (5.0 days vs. 7.0 days, respectively). The hospitalization time of the deterioration group was longer than that of the stable group (median 17.5 days vs. 12.0 days). All the basic features are shown in Table 1.

**Table 1:** Demographic and clinical characteristics of stable and deterioration patients

	All patients *N* = 239	Stable *N* = 177	Deterioration *N* = 62	*P* value
Characteristics				
Age (years) [median (SD)], n (%)	45.0 (15.9)	41.5 (14.6)	54.8 (15.5)	< 0.001
≤ 35	71 (29.7%)	64 (36.2%)	7 (11.3%)	< 0.001
35–45	53 (22.2%)	41 (23.2%)	12 (19.4%)	
45–55	61 (25.5%)	46 (26.0%)	15 (24.2%)	
> 55	54 (22.6%)	26 (14.7%)	28 (45.2%)	
Gender, n (%)				0.956
Female	113 (47.3%)	83 (46.9%)	30 (48.4%)	
Male	126 (52.7%)	94 (53.1%)	32 (51.6%)	
Imput case (close contact with Wuhan), n (%)				0.981
No	141 (59.0%)	105 (59.3%)	36 (58.1%)	
Yes	98 (41.0%)	72 (40.7%)	26 (41.9%)	
Frequent breathing (cpm^※^) median [IQR]	20.0 [20.0–21.0]	20.0 [20.0–20.0]	20.0 [20.0–22.0]	< 0.001
Blood pressure on admission (mmHg) [median (SD)]				
Systolic pressure	124 (13.0)	123 (12.7)	125 (13.9)	0.295
Diastolic pressure	76.9 (9.80)	76.6 (9.67)	77.8 (10.2)	0.396
Symptoms on admission				
Temperature (°C) median [IQR], n (%)	38.0 [36.8–38.5]	37.8 [36.6–38.4]	38.3 [38.0–38.6]	< 0.001
≤ 37.3	65 (27.2%)	60 (33.9%)	5 (8.1%)	< 0.001
37.3–38.0	67 (28.0%)	51 (28.8%)	16 (25.8%)	
38.0–39.0	94 (39.3%)	61 (34.5%)	33 (53.2%)	
> 39.0	13 (5.4%)	5 (2.8%)	8 (12.9%)	
Dyspnea, n (%)				< 0.001
No	177 (74.1%)	161 (91.0%)	16 (25.8%)	
Yes	62 (25.9%)	16 (9.0%)	46 (74.2%)	
Cough, n (%)				0.041
No	55 (23.0%)	49 (27.7%)	6 (9.68%)	
Yes	184 (77.0%)	128 (72.3%)	56 (90.3%)	
Headache, n (%)				0.564
No	203 (85.3%)	152 (86.4%)	51 (82.3%)	
Yes	35 (14.7%)	24 (13.6%)	11 (17.7%)	
Fatigue, n (%)				< 0.001
No	157 (66.0%)	129 (72.9%)	28 (45.9%)	
Yes	81 (34.0%)	48 (27.1%)	33 (54.1%)	
Muscle soreness, n (%)				0.372
No	210 (87.9%)	158 (89.3%)	52 (83.9%)	
Yes	29 (12.1%)	19 (10.7%)	10 (16.1%)	
Gastrointestinal symptoms (nausea, vomiting, diarrhea), n (%)				0.007
No	201 (84.1%)	156 (88.1%)	45 (72.6%)	
Yes	38 (15.9%)	21 (11.9%)	17 (27.4%)	
Incubation period (days) median [IQR]	7.0 [5.0–9.0]	7.0 [6.0–9.0]	5.0 [4.0–7.8]	< 0.001
Length of hospital stay (days) median [IQR]	13.0 (9.0–18.0)	12.0 (9.0–17.5)	17.5 (12.0–23.0)	< 0.001
Comorbidities, n (%)				< 0.001
No	161 (67.4%)	137 (77.4%)	24 (38.7%)	
Yes	78 (32.6%)	40 (22.6%)	38 (61.3%)	
Number of comorbidities median [IQR]	0 [0–1]	0 [0–1]	1 [1, 2]	< 0.001
Respiratory system disease (COPD^§^, chronic bronchitis, emphysema, asthma and bronchiectasis), n (%)				0.036
No	223 (93.3%)	169 (95.5%)	54 (87.1%)	
Yes	16 (6.69%)	8 (4.52%)	8 (12.9%)	
Cardiovascular diseases, n (%)				0.015
No	227 (95.0%)	172 (97.2%)	55 (88.7%)	
Yes	12 (5.02%)	5 (2.82%)	7 (11.3%)	
Endocrine system disease and metabolic related diseases (diabetes, obesity, hyperlipidemia and hyperthyroidism), n (%)				< 0.001
No	209 (87.4%)	165 (93.2%)	44 (71.0%)	
Yes	30 (12.6%)	12 (6.78%)	18 (29.0%)	
Hypertension, n (%)				< 0.001
No	205 (85.8%)	162 (91.5%)	43 (69.4%)	
Yes	34 (14.2%)	15 (8.47%)	19 (30.6%)	
Malignant tumour, n (%)				> 0.999
No	237 (99.2%)	175 (98.9%)	62 (100%)	
Yes	2 (0.8%)	2 (1.1%)	0 (0.00%)	
Digestive disease (hepatitis B, drug-induced hepatitis, fatty liver), n (%)				0.364
No	225 (94.1%)	168 (94.9%)	57 (91.9%)	
Yes	14 (5.86%)	9 (5.08%)	5 (8.06%)	

Continuous variable data are presented as median (SD or interquartile ranges, IQR). Classified variable data are presented as n (%). Unless otherwise stated, the Mann–Whitney U test is used for the continuous variable, the χ^2^ test, or the Fisher’s exact test for the categorical variable

^※^*cpm* counts per minute

^§^*COPD* chronic obstructive pulmonary disease

### Laboratory outcomes

The laboratory data showed significant differences in several blood tests between the deterioration and stable groups. For example, the deterioration group showed an increase in the following median compared with the stable group: neutrophilic-lymphocyte ratio (NLR, 5.30 vs. 3.02), myoglobin (91.2 vs. 55.0), creatine kinase (90.0 vs. 73.0), creatine kinase MB (14.0 vs. 12.0), blood urea nitrogen (4.77 vs. 3.90), lactate dehydrogenase (LDH, 255 vs. 203), D-dimer (0.42 vs. 0.25), C-reactive protein (CRP, 30.4 vs. 5.8), blood glucose (7.20 vs. 5.60), and CT score (10.5 vs. 3.0), as well as a decrease in the lymphocyte count (0.74 vs. 1.12), platelet count (151 vs. 202) and albumin (37.0 vs. 39.9), respectively (Table 2).

**Table 2:** Laboratory findings of all patients on admission

	All patients *N* = 239 Median [IQR]	Stable *N* = 177 Median [IQR]	Deterioration *N* = 62 Median [IQR]	*P* value
White cell count (× 10^9^/L)	5.00 [3.90–6.60]	5.00 [3.90–6.50]	5.13[3.91–6.99]	0.458
Neutrophil count (× 10^9^/L)	3.58 [2.63–6.38]	3.51 [2.55–5.57]	4.14 [2.79–6.58]	0.088
Lymphocyte count (× 10^9^/L)	1.00 [0.73–1.43]	1.12 [0.82–1.60]	0.74 [0.48–0.95]	< 0.001
NLR^※^	3.28 [2.16–6.16]	3.02 [1.95–5.17]	5.30 [3.09–9.14]	< 0.001
Platelet count (× 10^9^/L)	189 [139–241]	202 [152–246]	151 [124–208]	0.001
Haemoglobin (g/L)	133 [122–146]	135 [124–147]	130 [120–144]	0.194
D-dimer (mg/L)	0.27 [0.15–0.49]	0.25 [0.14–0.43]	0.42 [0.18–0.71]	0.002
Albumin (g/L)	39.0 [36.0–42.0]	39.9 [38.0–43.0]	37.0 [33.5–40.0]	< 0.001
Myoglobin (ng/mL)	60.8 [35.5–108]	55.0 [34.9–106]	91.2 [49.0–124]	0.003
C-reactive protein (mg/L)	8.30 [3.40–22.2]	5.80 [2.29–10.6]	30.4 [12.3–61.2]	< 0.001
Creatine kinase (U/L)	78.0 [51.0–110]	73.0 [51.0–101]	90.0 [56.5–168]	0.007
Creatine kinase MB (U/L)	12.7 [9.00–17.0]	12.0 [9.00–16.3]	14.0 [11.4–18.2]	0.020
Lactate dehydrogenase (U/L)	213 [169–268]	203 [161–244]	255 [212–337]	< 0.001
Blood urea nitrogen (mmol/L)	4.06 [3.08–5.10]	3.90 [3.00–5.00]	4.77 [3.58–5.45]	0.004
Creatinine (μmol/L)	70.0 [58.6–79.2]	69.0 [56.0–79.0]	72.3 [62.0–80.8]	0.111
Procalcitonin (ng/mL)	0.04 [0.03;0.10]	0.04 [0.03–0.10]	0.06 [0.04–0.10]	0.054
Blood Glucose (mmol/L)	5.80 [5.20–7.68]	5.60 [4.97–6.80]	7.20 [5.62–9.50]	< 0.001
Total bilirubin (mmol/L)	9.70 [7.60–15.1]	9.70 [7.60–14.8]	9.75 [7.62–15.8]	0.672
Direct bilirubin (mmol/L)	3.92 [3.00–5.45]	4.00 [3.00–5.54]	3.74 [3.12–5.38]	0.987
Semi-quantitative chest CT Score	4.00 [2.00–7.75]	3.00 [2.00–5.00]	10.5 [7.25–14.0]	< 0.001

Continuous variable data are presented as median (SD or inter quartile ranges, IQR). Unless otherwise stated, the Mann–Whitney U test is used for the continuous variable, the χ^2^ test, or the Fisher’s exact test for the categorical variable

^※^*NLR* neutrophil-to-lymphocyte ratio

### Predictor selection

The 44 variables measured at admission (Tables 1, 2) were included in the LASSO regression (Additional file 1: Fig. S1). A total of 9 variables with non-zero coefficients were obtained, including the dyspnea, incubation period, number of comorbidities, age, lymphocyte count, D-dimer, CRP, blood glucose, and CT score. In the univariate regression analysis (Table 3), all the variables were independent risk factors for deterioration (*P* < 0.05). However, when all the variables were incorporated in the logistic regression model, dyspnea, incubation period, CRP, and CT score were correlated with the risk of deterioration (*P* < 0.05). Those might be related to the existence of confounding or intermediate variables. Then, according to CIE, to adjust the independent variable blood glucose, age, and lymphocyte count in the regression model, it was found that the OR value for dyspnea was less than 10%, while that for adjusting latency, complications, D-dimer, CRP and CT scores was more than 10% (Additional file 1: Table S1). Furthermore, combined with the clinical and published studies [6, 7], the factors of dyspnea, incubation period, number of comorbidities, D-dimer, CRP and CT score were selected as risk model variables.

**Table 3:** Univariable and multivariable logistics regression models on the risk of deterioration

Variables	Univariable		Multivariable	
	Odds ratio (95% CI)	*P* value	Odds ratio (95% CI)	*P* value
Dyspnea	28.93 (13.44–62.26)	< 0.001	4.89 (1.53–15.80)	0.007
Incubation period (days)	0.83 (0.75–0.92)	< 0.001	0.83 (0.68–0.99)	0.049
Number of comorbidities	2.52 (1.81–3.52)	< 0.001	1.76 (1.03–3.05)	0.039
Age (years)	1.06 (1.04–1.08)	< 0.001		
Lymphocyte count (× 10^9^/L)	0.05 (0.02–0.14)	< 0.001		
D-dimer (mg/L)	6.17 (2.40–15.87)	< 0.001	7.05 (1.35–45.7)	0.029
C-reactive protein (mg/L)	1.09 (1.06–1.12)	< 0.001	1.06 (1.02–1.10)	0.007
Blood Glucose (mmol/L)	1.16 (1.07–1.27)	< 0.001		
Semi-quantitative chest CT score	1.70 (1.47–1.96)	< 0.001	1.50 (1.27–1.82)	< 0.001

*CI* confidential interval

### Construction of deterioration risk score

**Fig. 2:**
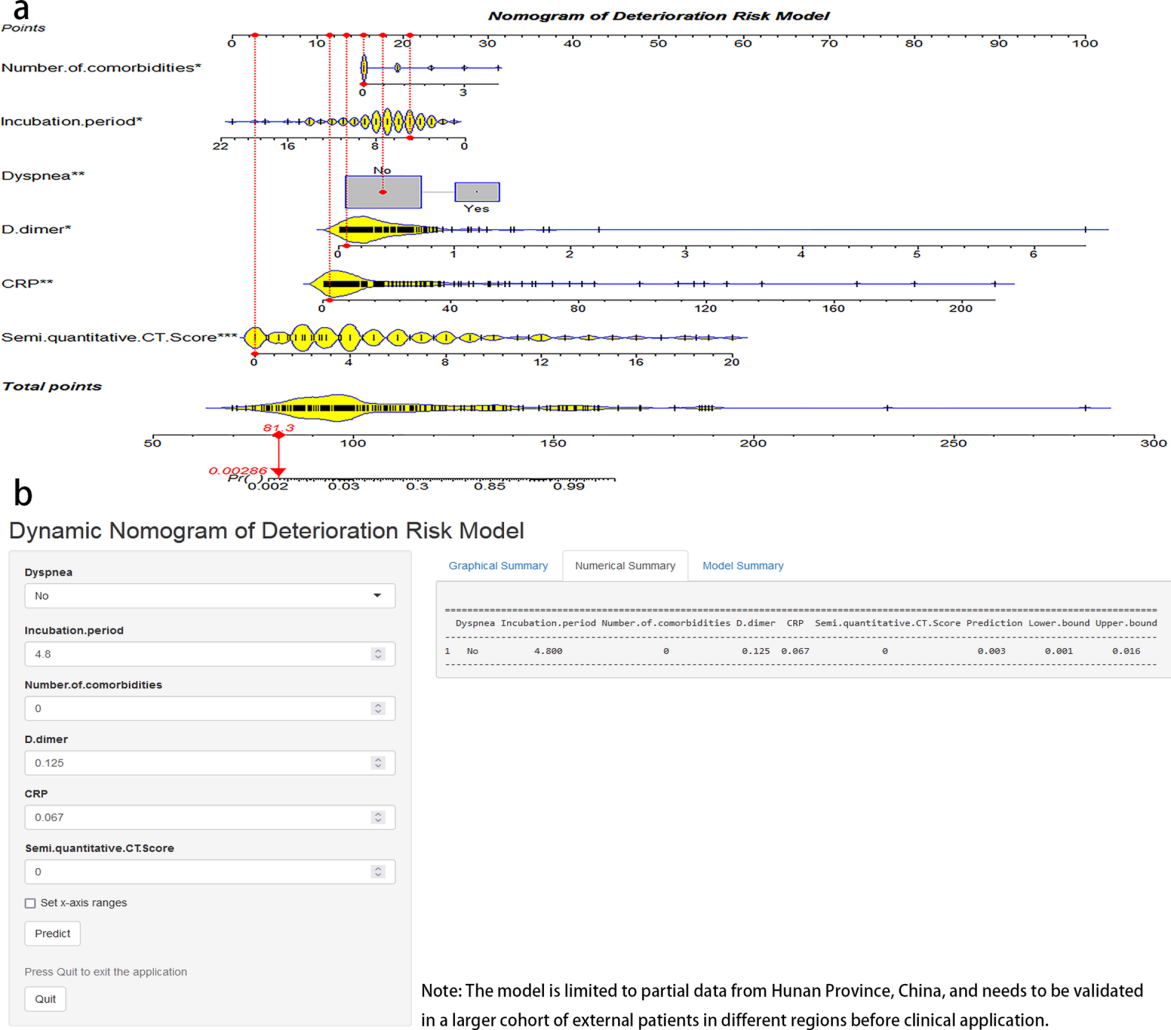
**a** Nomogram for predicting deterioration: each variable is projected onto line 1 to obtain the model. The sum of the scores is marked in the penultimate line, and a vertical line to the bottom line represents a final prediction from the risk score count. The case shown here is with an incubation period 4.8 days, no dyspnea, no number of comorbidities, D-dimer 0.128 mg/L, CRP 0.067 mg/L, and no CT lesions in the lungs, final score 81.3, predicted risk (pr) value 0.00286, low risk of deterioration. **b** The case calculated a relative predictive value of 0.003 (95% CI 0.001–0.016) according to the variable through the network counter. *CRP* C-reactive protein

In the multivariate analysis (Table 3), the logistic regression model identified the correlation between 6 variables and DRM-COVID-19. The factors of the incubation period (OR 0.83; 95% CI 0.68–0.99; *P* = 0.049) were negatively correlated, while the dyspnea (OR 4.89; 95% CI 1.53–15.80; *P* = 0.007), number of comorbidities (OR 1.76; 95% CI 1.03–3.05; *P* = 0.039), D-dimer (OR 7.05; 95% CI 1.35–45.7; *P* = 0.029), CRP (OR 1.06; 95% CI 1.02–1.1; *P* = 0.007) and CT score (OR 1.50; 95% CI 1.27–1.82; *P* < 0.001) were positively correlated. The model fits well (Hosmer–Lemeshow test, X^2^(8) = 7.0194, *P* = 0.53). A nomogram for DRM-COVID-19 containing the dyspnea, incubation period, number of comorbidities, D-dimer, CRP, and CT score was constructed (Fig. 2a). Furthermore, an online calculator based on the nomogram was developed, allowing the clinicians to automatically calculate the deterioration risk of COVID-19 patients (and 95% CI) (Fig. 2b), available online at (https://deterioration-risk-model-of-covid-19.shinyapps.io/DRMapp/).

### Risk model evaluation and internal validation

**Fig. 3:**
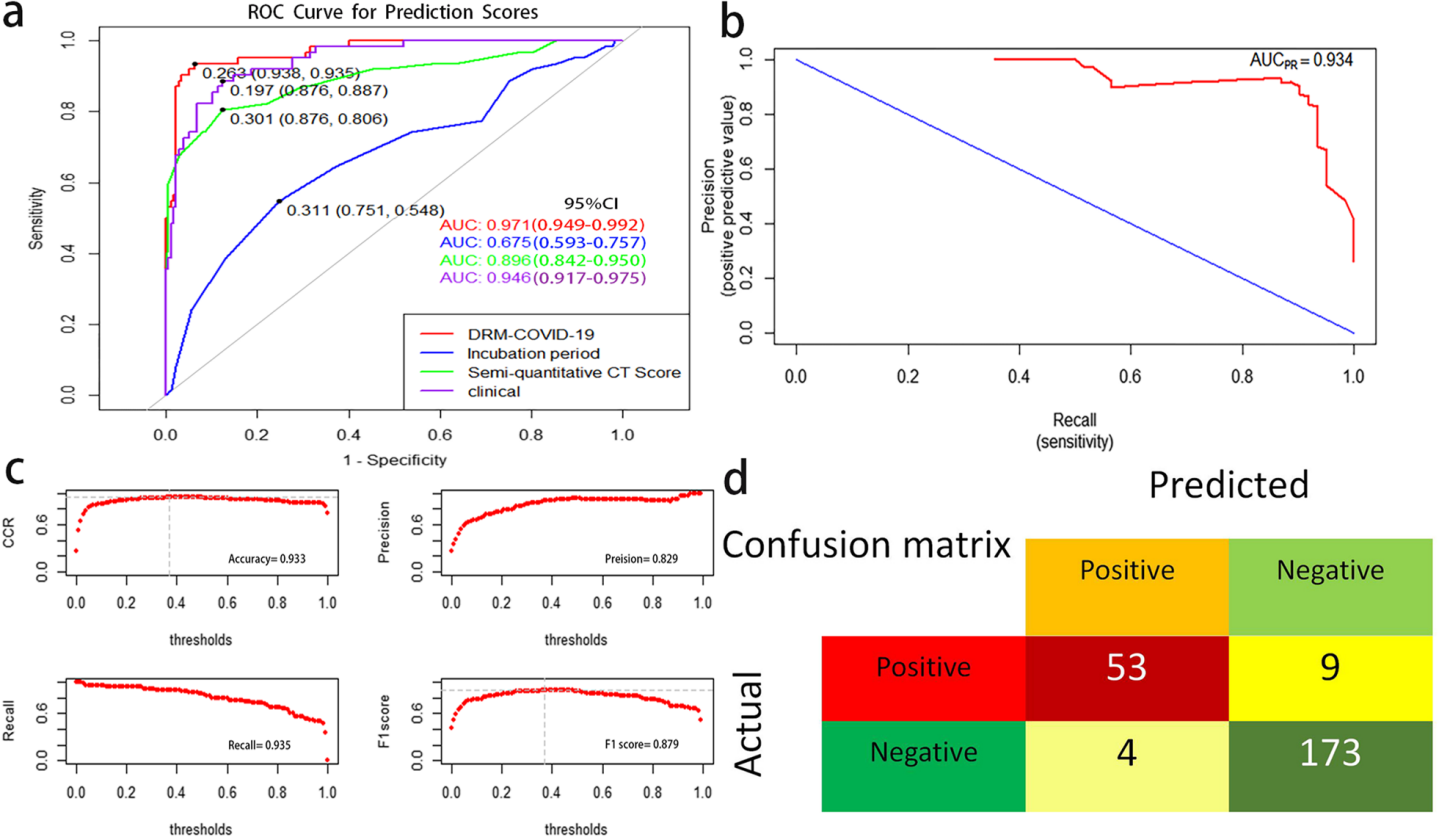
**a** Using receiver operating characteristic (ROC) curve and area under the receiver operating characteristic curve (AUC) to compare the discriminant ability of four models based on DRM-COVID-19, incubation period, clinical, and CT scores. **b** Due to the imbalance of the data set, the precision-recall (PR) curve was used further to evaluate the classification ability of the predictive model; AUC_PR_ (0.934) indicated the model performed well in predicting case classification. **c** The accuracy, precision, recall, and F1 score values were 0.933, 0.829, 0.935, and 0.879, respectively. **d** In the PR curve of DRM-COVID-19, the confusion matrix was constructed. *DRM-COVID-19* deterioration risk model of COVID-19

**Fig. 4:**
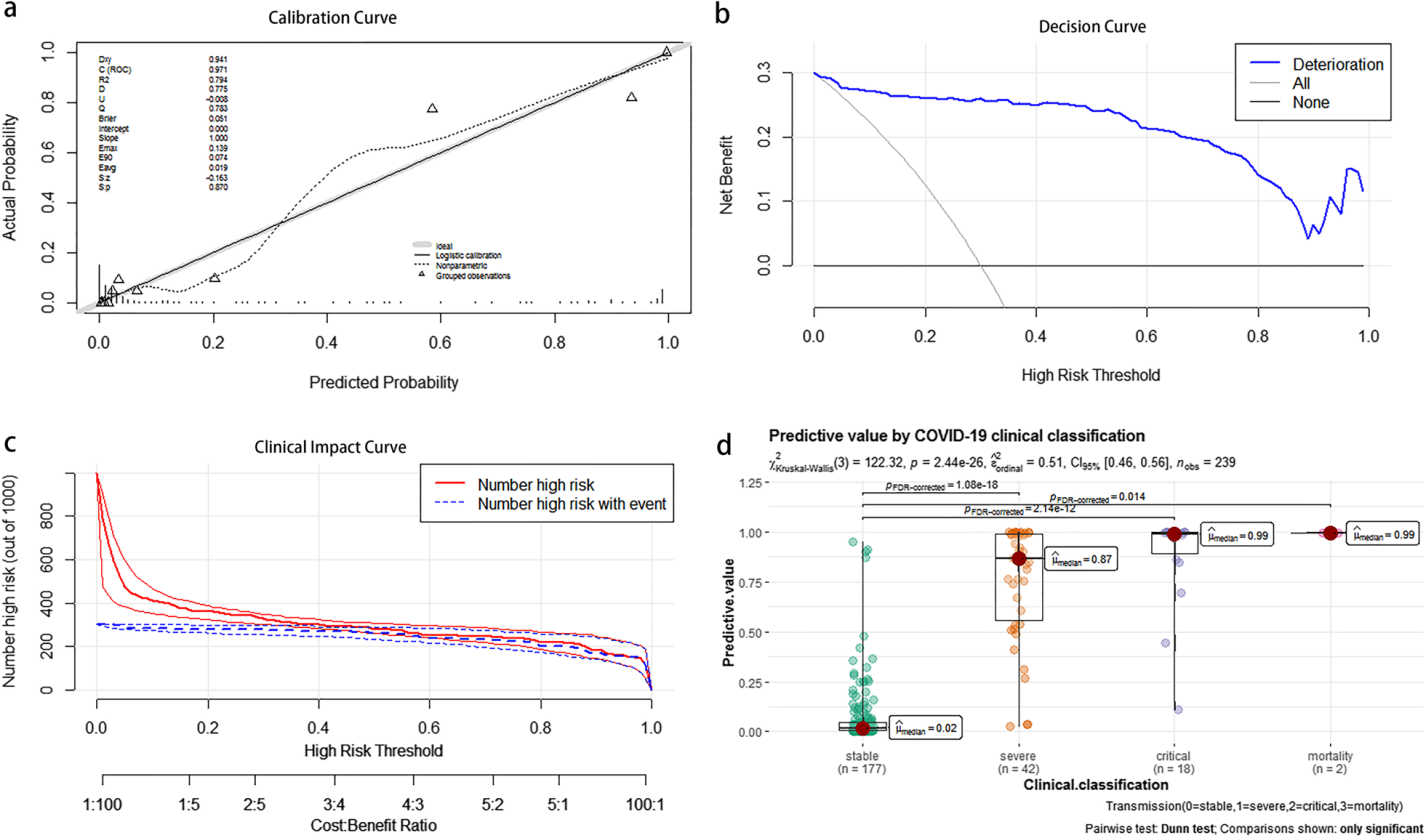
**a** The calibration curve showed high coherence between the predicted and actual probability of deterioration. **b** Decision curve of DRM-COVID-19; blue line: risk value. Light line: assume all patients are getting deterioration. Horizontal thin line: assumed no patient is getting deterioration. The chart shows the expected net benefit of each patient relative to the stability of any patient. The farther away the curve is from the horizontal thin line, the greater the clinical benefit. **c** Clinical impact curve of DRM-COVID-19 Of 1000 patients, the solid red line represents the total number of deterioration considered high-risk at each risk threshold. The blue dotted line shows how many people actually need to have deteriorated. **d** The scatter chart shows the predicted value distribution and median value of different severity cases. Meanwhile, the Kruskal–Wallis test showed a statistical difference between the stable and the other groups. *DRM-COVID-19* deterioration risk model of COVID-19

We obtained the deterioration risk scores in four models based on DRM-COVID-19, incubation period, clinical, and CT scores. The ROC curves were plotted for the above-mentioned models (Fig. 3a), and the AUC values were 0.971 (95% CI: 0.949–0.992; specificity 0.938, sensitivity 0.935), 0.675 (95% CI: 0.593–0.757; specificity 0.751, sensitivity 0.548), 0.946 (95% CI: 0.917–0.975; specificity 0.876, sensitivity 0.887), and 0.896 (95% CI: 0.842–0.950; specificity 0.876, sensitivity 0.806), respectively. Therefore, the discriminative ability, sensitivity, and specificity of DRM-COVID-19 for patients with high-risk deterioration showed better values than other models. The optimal cut-off value of the ROC curve for DRM-COVID-19 was 0.263. The area under the precision-recall curve (AUC_PR_ = 0.934) was provided by the PR curve, which showed the good classification performance of the model (Fig. 3b). The confusion matrix was constructed In the PR curve of DRM-COVID-19 (Fig. 3d). The accuracy, precision, recall, and F1 score values were 0.933, 0.829, 0.935, and 0.879, respectively (Fig. 3c). These results indicate that DRM-COVID-19 achieved positive performance in the deterioration and stable groups. Meanwhile, we also noted that the recall was higher than the precision, meeting the requirement of low misjudgment risk. As shown in Fig. 4, the model had good calibration for the COVID-19 deterioration prediction with no significant overestimation or underestimation (c-statistic = 0.971; R2 = 0.794). Measure precision by Brier score (0.051, 95% CI, 0.03–0.072) (Fig. 4a). The decision curve showed a good net benefit across the range of 0 to 100% (Fig. 4b). The clinical impact curve of DRM-COVID-19 among 1000 patients visually indicates the number of high-risk deteriorations (solid red line) versus the actual number of deterioration (dotted blue line, Fig. 4c). We performed internal validation of the logistic regression model and obtained the c-statistic 0.957 and R-squared 0.588 through bootstrap resampling for 1000 times. As a result, the prediction ability of the model is acceptable.

### Secondary outcomes

In the scatter chart (Fig. 4d), the Kruskal–Wallis test showed a statistical difference between the stable and deterioration groups (*P* < 0.001). Compared with the stable group, there was a statistical significance in the other three groups (*P* < 0.05). In contrast, the mortality group had a *P* value of 0.014, which may be related to the small number of cases. As the COVID-19 severity increased, the median predictive value of each clinical classification also showed an upward trend (stable, 0.02; severe, 0.87; critical, 0.99; mortality, 0.99), which suggests that the DRM-COVID-19 model has a predictive value for severity.

Univariate Cox regression analysis showed the optimal cut-off value of DRM-COVID-19 had an excellent predictive ability for deterioration within 15 days (HR 74.54 95% CI 26.81–207.2), and the Log-rank test showed significance *P* value < 0.0001 (Additional file 1: Fig. S2a). Schoenfeld Individual was calculated to test the proportional risk hypothesis test (*P* = 0.331) (Additional file 1: Fig. S2b). Similarly, the high-risk group spent more time in the hospital than the low-risk group (HR 20.68 95% CI 7.49–57.15, Log-rank test *P* < 0.0001, Additional file 1: Fig. S2c, d), and the case with a hospital stay of up to 50 days was a hemodialysis patient with a repeated nucleic acid test positive.

## Discussion

In this study, based on a COVID-19 multicenter retrospective cohort with 239 cases, we developed and internally validated a predictive model to help clinicians predict the deterioration risk of the patients upon admission, thus providing possible help for early triage and management of these patients. The internal verification indicated that the proposed DRM-COVID-19 model fits well. In the predictive model, the factors of dyspnea, incubation period, number of comorbidities, D-dimer, CRP and CT score were the most significant risk factors. These parameters are routinely measured for COVID-19 inpatients. At the same time, we prepared a network platform, which is convenient for the clinicians to operate.

Since the emergence of the COVID-19 pandemic, many predictive models concerning the diagnosis and prognosis have emerged [5, 9, 16, 29–31]. In the development of COVID-19 prediction models, artificial intelligence, including machine learning and deep learning, has been widely used to improve the accuracy and expansibility of the prediction models [32]. Lasso regression and Logistic regression used in our research belong to the machine learning algorithms. The most important thing of a development regression model is to strike a balance between the influencing factors and the control bias to avoid over-fitting and under-fitting. Specifically, when datasets have few events, penalty regression is superior to standard regression and provides better prediction [33]. In this study, we adopted Lasso regression (lambda.1se) to obtain the nine optimal variables due to the data with few events. Usually, the events per variables ratio should be 10 or more [33, 34]. Sixty-two cases in the cohort met the primary outcome of deterioration, so the variables of the DRM-COVID-19 should not exceed six. The conventional stepwise regression is passive to eliminate some variables through covariable coefficients, quickly leading to invalid estimation and predictive effects [21, 22]. The change-in-estimate (CIE) represents a standard method in epidemic disease studies. Therefore, to positively control confounders, we adopted the measure of combining CIE with the background knowledge of COVID-19 [6] to select and adjust nine variables. Finally, we constructed predictive models including the dyspnea, incubation period, number of comorbidities, D-dimer, CRP, and CT score. This compound parameter screening method is an exciting attempt in our study, which achieved an excellent predictive performance (AUC 0.971, 95% CI 0.949–0.992). Moreover, the calibration curve showed high coherence between the predicted and actual deterioration probability. The clinical decision-making curve and clinical impact curve show that when DRM-COVID-19 is used to determine whether the patient has the risk of being hospitalized or not, better clinical benefits than "full deterioration" or "non-deterioration" will be obtained.

For unbalanced datasets, the ROC curve is considered to be deceptive to the interpretability and reliability of the model classification performance to a certain extent [35]. Therefore, other evaluation methods are often introduced into machine learning [14]. The obtained results of the PR curve (AUC_PR_ 0.934), accuracy (0.933), precision (0.829), recall (0.935) and F1 score (0.879) showed a good classification performance of RDS-COVID-19. In our predicted model, recall is higher than precision to avoid missing the cases that may aggravate.

The variables commonly found in published literature, such as dyspnea, number of comorbidities, D-dimer, and CRP, were associated with clinical endpoints requiring mechanical ventilation, ICU admission, and mortality [5–7]. These variables were also included in our risk model. Since the COVID-19 pandemic, numerous diagnostic and prognostic models have emerged [36]. Different prediction models have different prediction factors [5, 9]. Even with similar research purposes [9–13], the predictors might not be identical. Reasons might include different endpoints, different populations, or different study methods. In our study, the variables screened by LASSO regression included age and lymphocyte count, which were common risk factors in the COVID-19 prediction model [9], but were not included in our model, which may also be related to the reasons mentioned earlier.

We found that the longer the incubation period, the lower the risk of developing into severe or critical illness, following the previously published results [37]. The main reason for the short incubation period is the more significant virus load in the body. Respiratory viruses induce the immune response through inflammatory mediators and cytokines, leading to clinical symptoms, thus determining the incubation period [38]. Pneumonia caused by SARS-CoV-2 is also related to the immune response. The shorter incubation period was associated with more pulmonary exudate lesions [37], and a greater risk for aggravation or hospitalization. Therefore, the incubation period is an independent risk factor for the DRM-COVID-19 model.

Another prominent variable was the CT score. The CT score of the deterioration group (mean value ± SD, 10.8 ± 5.0) was significantly higher than the stable group (3.5 ± 2.5, *P* < 0.0001). The study of Francone and colleagues [16] showed that the CT score of the critical group (20.3 ± 3) and the severe group (17.4 ± 3.1) were significantly higher than those of the mild group (8.7 ± 4, *P* < 0.001). The CT score of the deterioration group in our study was lower than that of the above-mentioned severe group and slightly higher than that of the mild group. These differences may be related to the different detection times of lung CT. In our study, lung CT was detected at admission or before aggravation. Another study showed that the cut-off value of the CT score was 7, with good sensitivity (80.0%) and excellent specificity (82.8%) [17]. Interestingly, the CT values of our deterioration group ranged from 7.25 to 14.0 in the quartile. The CT score can act as an independent risk factor for the deterioration of COVID-19.

In addition, the scatter diagram was prepared based on the DRM-COVID-19 predictive models of the stable (mild/moderate), severe, critical and mortal groups. The predictive model could distinguish the stable group well from the other groups (*P* < 0.05). With 0.25 as the distinguishing line on the violin chart, 93.5% of the deterioration cases could be distinguished, 92.9% of the severe cases could be accurately predicted, and 95% of the critical cases (including the mortality cases) could be identified. Interestingly, the cutoff value of the ROC curve was 0.263, with a sensitivity of 93.5% and a specificity of 93.8%. Therefore, when the DRM-COVID-19 network calculator is used for triage of hospitalized patients with a score greater than 0.263, we need aggressive treatment to prevent further deterioration. The high-risk and low-risk groups based on the optimal cutoff value also achieved excellent results in predicting the risk of deterioration within 15 days and the length of hospitalization.

## Limitations

This study has some limitations. First, the dataset included partial data collected from one province. Due to the relatively small number of cases, LASSO regression and binary logistics did not follow a training set and testing set to evaluate the generalization ability of the model. Therefore, we adopted a tenfold cross-validation method in LASSO regression and 1000 times bootstrap resampling internal verification in binary logistic regression. There was also not enough data as a validation set for external validation. Those would increase the risk of overfitting the model. Second, our prediction model is only based on Chinese data from the first wave of the epidemic, and the treatment and care of patients at that time were not homogeneous and standardized. It should be verified whether it applies to other countries or regions or currently the main variants cases (delta-variant or omicron-variant). Those are essential steps in verifying the generalization ability of the model. Third, due to the rapid control of the epidemic, we could not collect more data, especially positive data. Although good results were obtained in the analysis of unbalanced data, we were still cautious in the triage of the cases. Finally, the control of confounding factors and the elimination of intermediate variables still pose the risk of misjudgment.

## Conclusion

In this study, we used CIE to screen variables based on the Lasso regression to avoid the risk of over-fitting for the prediction model due to the small sample size. We first developed a COVID-19 aggravation risk prediction model based on the incubation period, clinical, and chest images. The predicted value of DRM-COVID-19 can effectively predict the risk of deterioration within 15 days. The prediction model can triage each symptomatic COVID-19 patient and ensure the appropriate level of care according to the risk of deterioration, thus reducing deterioration rate, optimizing the medical resources and alleviating medical stress.

## Supplementary Information

**Additional file 1.** **Figure S1:** LASSO regression screening variables. **Table S1:** The Change-in-Estimate (CIE) is used for variable selection. **Figure S2:** COX regression.

## Data Availability

All data relevant to this study are included in the article or uploaded as supplementary information. The datasets used and/or analyzed during the current study are available from the corresponding author on reasonable request.
