# Supplementary material for: Incubation period, clinical and lung CT features for early prediction of COVID-19 deterioration: development and internal verification of a risk model
Source: BMC Pulm Med. 2022 May 12;22:188. doi: 10.1186/s12890-022-01986-0 (PMC9095818; doi:10.1186/s12890-022-01986-0)
Supplement: Supplementary file 1 — Additional file 1. Figure S1: LASSO regression screening variables. Table S1: The Change-in-Estimate (CIE) is used for variable selection. Figure S2: COX regression. [file 12890_2022_1986_MOESM1_ESM.pdf]

Supplementary Online Content

1. Supplemental Fig 1. LASSO regression screening variables.
2. Supplemental Table 1. The Change-in-Estimate（CIE）is used for variable selection.
3. Supplemental Fig 2. COX regression

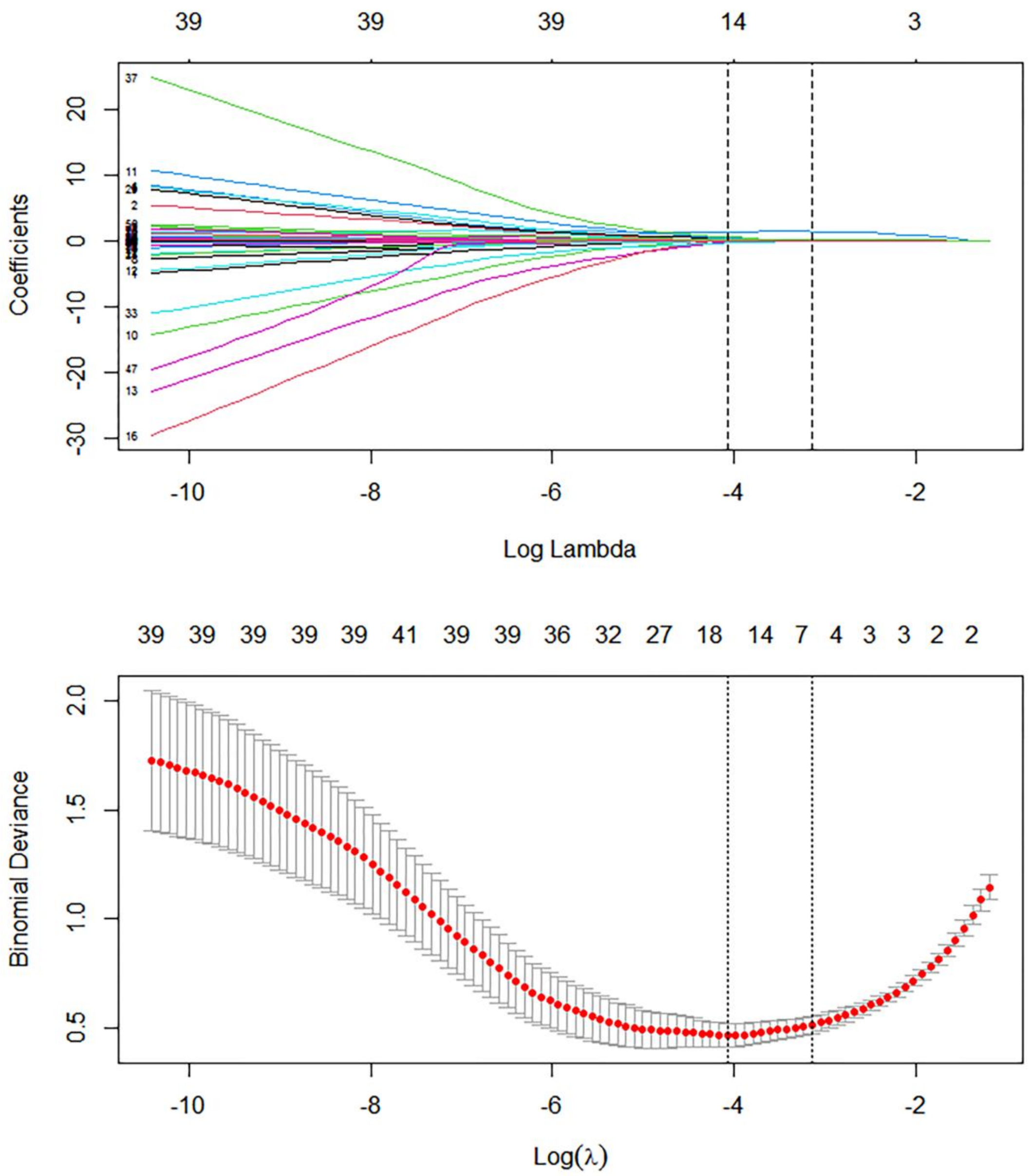

Supplemental Fig 1 Screening demographic and clinical characteristic variables with the Least Absolute Shrinkage and Selection Operator(LASSO) regression. Draw dotted vertical lines at the optimal value with a standard error of the minimum criterion(lambda.min) and the minimum criterion (1-SE criterion,lambda.1se). The variables selected by lambda.1se are used in logistics regression.

Supplemental Table 1. The Change-in-Estimate（CIE）is used for variable selection.

Odds ratio / Variables	First	Second	Third	Fourth	Fifth	Sixth	Seventh	Eighth	Ninth	Tenth
Dyspnea	4. 83	4. 85	4. 50	5. 52	4. 89	5.58	13. 21	5. 91	6. 81	6. 69
Incubation period	0. 82	0. 82	0. 83	0. 85	0. 83		0. 82	0. 85	0. 82	0. 86
No.of comorbidities	1. 40	1. 44	1. 81		1. 76	1.67	1. 88		1. 82	1. 80
Age	1. 03	1.03								
Lymphocyte count	0. 34	0. 34	0. 33	0.37						
D-dimer	6. 22	6. 38	6. 65	7.47	7.05	7.13	2.52	7.92		5.91
C-reactive protein	1. 05	1. 05	1. 05	1. 05	1. 06	1.05	1.07	1.06	1. 05	
Blood Glucose	1. 03									
Semi-quantitative chest CT score	1. 42	1. 44	1. 45	1.47	1.5	1.51		1.51	1.45	1.61

The gradual elimination of blood glucose, age and lymphocyte count had little influence on the OR value(<10%) of dyspnea in the multivariable logistic regression model, and the subtraction of incubation period, dyspnea, number of comorbidities, D-dimer, CRP and CT score successively had an influence of >10% on OR value. Therefore, the remaining six variables were included in the final risk score. CRP: C-reactive protein.

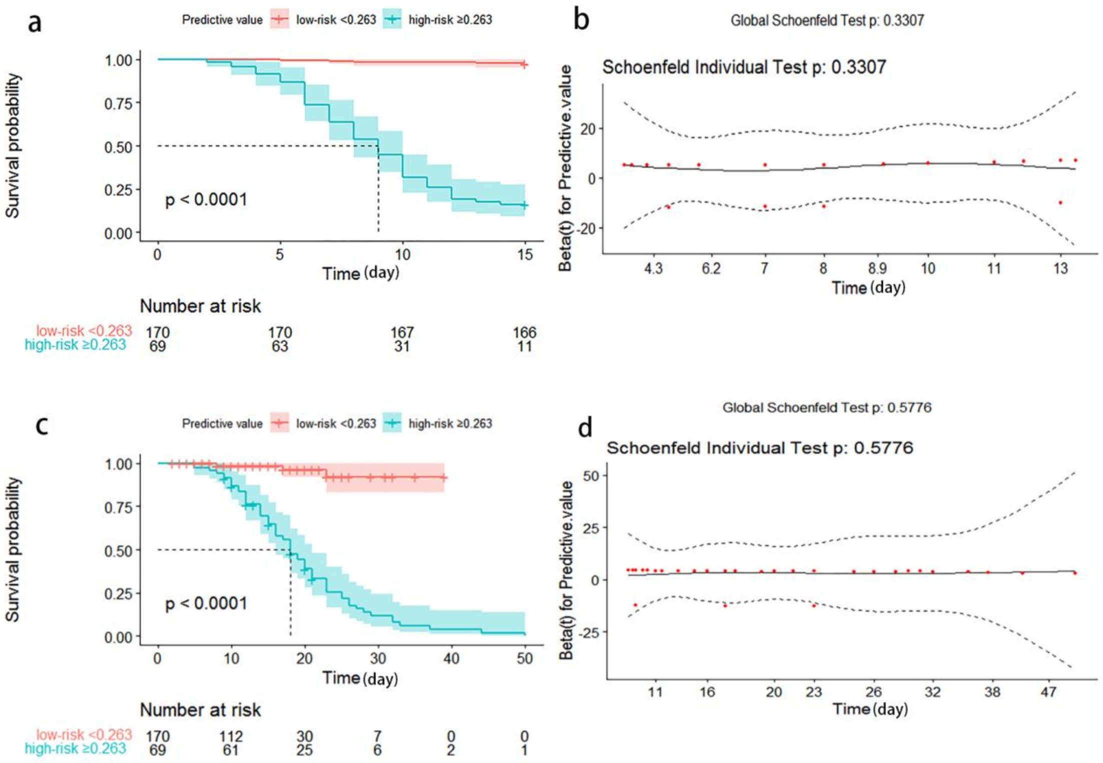

Supplemental Fig 2 a Univariate COX regression analysis showed that the optimal cut-off value of DRM-COVID-19 had an excellent predictive ability for deterioration within 15 days (74.54 95%CI 26.81-207.2, Log-rank tests P-value < 0.0001). b Schoenfeld Individual test diagnostic diagram visualization proportional risk hypothesis test, P-value 0.3307.c the high-risk group spent more time in the hospital than the low-risk group (HR 20.68 95%CI 7.49-57.15, Log-rank test $p<0.0001$). d Schoenfeld Individual test diagnostic diagram visualization proportional risk hypothesis test, P-value 0.5776. DRM-COVID-19: deterioration risk model of COVID-19.
